# SIK2 represses AKT/GSK3β/β‐catenin signaling and suppresses gastric cancer by inhibiting autophagic degradation of protein phosphatases

**DOI:** 10.1002/1878-0261.12838

**Published:** 2020-11-20

**Authors:** Xiao‐man Dai, Yan‐hui Zhang, Xiao‐han Lin, Xiao‐xing Huang, Yi Zhang, Chao‐rong Xue, Wan‐nan Chen, Jian‐xin Ye, Xin‐jian Lin, Xu Lin

**Affiliations:** ^1^ Key Laboratory of Gastrointestinal Cancer (Fujian Medical University) Ministry of Education Fuzhou China; ^2^ Fujian Key Laboratory of Tumor Microbiology Fujian Medical University Fuzhou China; ^3^ Department of Gastrointestinal Surgery The First Affiliated Hospital of Fujian Medical University Fuzhou China

**Keywords:** AKT/GSK3β/β‐catenin signaling, autophagy, gastric cancer, protein phosphatases, SIK2

## Abstract

Salt‐inducible kinase 2 (SIK2) is an important regulator in various intracellular signaling pathways related to apoptosis, tumorigenesis and metastasis. However, the involvement of SIK2 in gastric tumorigenesis and the functional linkage with gastric cancer (GC) progression remain to be defined. Here, we report that SIK2 was significantly downregulated in human GC tissues, and reduced SIK2 expression was associated with poor prognosis of patients. Overexpression of SIK2 suppressed the migration and invasion of GC cells, whereas knockdown of SIK2 enhanced cell migratory and invasive capability as well as metastatic potential. These changes in the malignant phenotype resulted from the ability of SIK2 to suppress epithelial–mesenchymal transition via inhibition of AKT/GSK3β/β‐catenin signaling. The inhibitory effect of SIK2 on AKT/GSK3β/β‐catenin signaling was mediated primarily through inactivation of AKT, due to its enhanced dephosphorylation by the upregulated protein phosphatases PHLPP2 and PP2A. The upregulation of PHLPP2 and PP2A was attributable to SIK2 phosphorylation and activation of mTORC1, which inhibited autophagic degradation of these two phosphatases. These results suggest that SIK2 acts as a tumor suppressor in GC and may serve as a novel prognostic biomarker and therapeutic target for this tumor.

## Graphical abstract

Salt‐inducible kinase 2 (SIK2) functions as a tumor suppressor in gastric cancer (GC). Activation of mTORC1 by SIK2 inhibits autophagic degradation of the PP2A and PHLPP2 protein phosphatases, thereby increasing dephosphorylation and inactivation of AKT/GSK3β/β‐catenin signaling. Our results highlight SIK2 as a potential therapeutic target in GC.

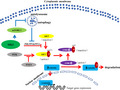

**Abbreviations**
AMPK: AMP‐activated protein kinase
Co‐IP: co‐immunoprecipitation
EMT: epithelial–mesenchymal transition
GAPDH: glyceraldehyde‐3‐phosphate dehydrogenase
GC: gastric cancer
GEO: Gene Expression Omnibus
H&E: hematoxylin and eosin
IHC: immunohistochemistry
mTOR: mechanistic target of rapamycin
NC: negative control
PHLPP: PH domain leucine‐rich repeat protein phosphatase
PP2A: protein phosphatase 2A
qRT‐PCR: quantitative real‐time polymerase chain reaction
SIK2: salt‐inducible kinase 2
TCF/LEF: T cell factor/lymphoid enhancer‐binding factor
TCGA: The Cancer Genome Atlas

## 1 Introduction

According to GLOBOCAN 2018 data, gastric cancer (GC) represents the fifth highest incidence among all human malignancies, with over a million new cases being diagnosed globally each year [1]. It is also the third driving cause of cancer deaths around the world, following lung and colorectal cancer in overall mortality [1]. Although critical global endeavors have been made in the prevention and treatment of GC in recent decades, the clinical outcome stays inauspicious because of the fact that a majority of GC patients present at an advanced disease at initial diagnosis, thus missing the best timing for radical surgery, and that current standard chemotherapy only gives a moderate survival advantage. Therefore, identification of crucial molecular signaling pathways associated with gastric carcinogenesis and progression would help in early diagnosis and possibly offer a rational design for targeted therapies to advanced GC.

AMP‐activated protein kinase (AMPK) is a key mediator of cellular metabolism and energy homeostasis. Its relevance to cancer biology lies in the fact that AMPK is interconnected with signaling networks involving established tumor suppressors such as LKB1 [2, 3], TSC2 [4] and p53 [5]. It has been assumed that activation of *AMPK* could impede the development of cancer via reprogramming cellular metabolism and hitting the critical components needed for tumor progression [6]. However, activation of *AMPK* can also provide a survival advantage to cancerous cells via regulation of cellular metabolic plasticity to adapt to metabolic stress [7]. Therefore, a pro‐ or anti‐tumorigenic role of AMPK in cancer may well be dependent on tumor tissue‐specific context. The salt‐inducible kinase (SIK) is a serine/threonine protein kinase belonging to a family of AMPK. Among the three isoforms of SIK family [namely, salt‐inducible kinase (SIK)1, SIK2 and SIK3], SIK2 is of particular interest due to involvement in several major signaling pathways such as the PI3K‐AKT‐mechanistic target of rapamycin (mTOR) pathway, the LKB1‐HDAC axis, the Hippo‐YAP pathway and the cAMP‐PKA axis, all of which are related to tumorigenesis and tumor progression [8]. Similar to AMPK, SIK2 has a dual role functioning as oncogenic driver or tumor suppressor in different cancer cell types [9]. SIK2 has been shown to act as a potential tumor promoter in ovarian [9, 10], prostate [11], osteosarcoma [12] and colorectal [13] cancers. In contrast, SIK2 may serve as a tumor suppressor in breast cancer [14] and pancreatic ductal adenocarcinomas [15]. However, irrespective of this dichotomy, the function and mechanism of SIK2 in GC tumorigenesis and progression are yet to be elucidated.

In the present study, we report that SIK2 expression was significantly downregulated in GC tissues as compared with their adjacent normal tissues, and reduced SIK2 expression predicted poor prognosis of the patients. SIK2 functioned to sustain an epithelial phenotype and loss of SIK2 promoted epithelial‐mesenchymal transition (EMT) via regulation of AKT/GSK3β/β‐catenin signaling pathway. Moreover, our study demonstrated that SIK2 promoted phosphorylation and activation of mTORC1, leading to inhibition of autophagic degradation of the protein phosphatases PHLPP2 and PP2A, which consequently enhanced AKT dephosphorylation and inactivation. Thus, our data identify SIK2 as a novel tumor suppressor in GC and provide important insights into the mechanisms of GC development and progression as well as highlighting the potential target for therapeutic interventions.

## 2 Materials and methods

### 2.1 Cell culture

Two human GC cell lines, AGS and MGC803, were purchased from the Type Culture Collection of the Chinese Academy of Sciences (Shanghai, China) and cultured respectively in Dulbecco’s modified Eagle Medium/F12 and RPMI‐1640 (Gibco BRL, Gaithersburg, MD, USA) medium supplemented with 10% FBS. Cell lines were tested routinely for the absence of mycoplasma contamination by a MycoAlert detection kit (Lonza, Walkersville, MD, USA) and authenticated by short tandem repeat analysis every 6 months.

### 2.2 Tissue microarray and immunohistochemistry

Human GC samples and their corresponding adjacent gastric tissues were collected from patients who underwent surgical resection without receiving preoperative chemotherapy at Fujian Medical University Union Hospital (Fuzhou, China) from 2010 to 2011. This study conformed to the standards set by the Declaration of Helsinki and was approved by the Ethics Committee of Fujian Medical University. Written informed consent was acquired from all participants. A tissue microarrayer (Outdo Biotech, Shanghai, China) was used to construct a tissue microarray block that contained 180 GC and paired adjacent normal tissues. Immunohistochemistry (IHC) of tissue microarray slides was performed using an anti‐SIK2 antibody (Abcam, Cambridge, UK). A 5‐tiered scale was applied to determine the degree of SIK2 staining according to the average percentage of positively stained cells (0: ≤ 5% positive cells; 1: 5–25% positive cells; 2: 26–50% positive cells; 3: 51–75% positive cells; 4: 76–100% positive cells), which was then multiplied by the staining intensity (0, no staining; 1, weak staining, light yellow; 2, moderate staining, yellow‐brown; and 3, strong staining, brown) to obtain a final score of 0 to 12. A final score of < 4 was categorized as low SIK2 expression and a score > 4 as high SIK2 expression.

### 2.3 Western blot analysis

Proteins were isolated by using RIPA buffer (Beyotime Biotechnology, Shanghai, China) containing protease inhibitor cocktail (Roche Diagnostics Co., Indianapolis, IN, USA), separated by electrophoresis in SDS/PAGE gel, and then transferred to poly(vinylidene difluoride) membrane (GE Healthcare, München, Germany). The membrane was blocked in PBS‐T buffer containing 5% milk/BSA for 2 h and incubated with a primary antibody overnight at 4 °C. After washing three times, the membrane was incubated with horseradish peroxidase‐labeled HRP‐coupled secondary antibody for 1 h and visualized with an ECL detection reagent (BeyoECL Star; Beyotime). The primary antibodies are listed in Table S1.

### 2.4 Quantitative real‐time PCR

Total RNA was extracted from cell lines or tissues using Qiagen RNeasy kit following the manufacturer’s instructions. First‐strand cDNA was synthesized using total RNA with Revert Aid First Strand cDNA Synthesis (Fermentas, Vilnius, Lithuania). Quantitative real‐time PCR was performed in an Mx3000P Real‐Time PCR System (Agilent Technologies, Santa Clara, CA, USA) with the SYBR Premix Ex TaqTM kit (Takara, Tokyo, Japan). The β‐actin gene served as the reference gene, and relative mRNA levels were calculated by the $2^{‐\Delta \Delta C_{t}}$ method. Primer sequences are shown in Table S2).

### 2.5 Plasmid construction and establishment of stably transduced GC cell lines

The open reading frame of human *SIK2* (Gene ID: 23235) was cloned into the lentiviral expression vector pCDH (Invitrogen, Carlsbad, CA, USA). The recombinant plasmid pSIK2, lentivirus packaging plasmids pMDL, p‐VSV‐G and p‐REV (Invitrogen) were co‐transfected into 293T at 80% confluence in 10‐cm culture dishes. The supernatant was collected and filtrated through 0.45‐µm filters and then added into the culture of GC cells (AGS and MGC803) in 6‐cm culture dishes. Two days after incubation, 1 µg·mL^−1^ puromycin (Invitrogen) was added to select stably transduced cells for 7 days. Stable SIK2 overexpressed clones (AGS‐pSIK2 or MGC803‐pSIK2) and the empty vector pCDH‐transfected cells as negative controls (NC) (AGS‐pCDH or MGC803‐pCDH) were used for subsequent experiments. To generate SIK2 knocked down clones, two short hairpin (sh)RNA sequences targeting different regions of SIK2 were synthesized and cloned into pSUPER‐retro‐puro plasmid (Oligoengine). The recombinant plasmid or NC vector ligated with a scrambled‐base hairpin oligo was co‐transfected with packaging plasmids pIK (Invitrogen) into 293T cells. The supernatants were collected and used to infect AGS or MGC803 cells. The puromycin‐resistant clones were expanded into cell lines as SIK2 knockdown cells (AGS‐psh1SIK2, AGS‐psh2SIK2, MGC803‐psh1SIK2, MGC803‐psh2SIK2) or the control cells (AGS‐pSUPER or MGC803‐pSUPER). To rescue SIK2 expression in the sh1SIK2 knockdown MGC803 cells, six synonymous point mutations were introduced into the sh1SIK2 target regions in the full‐length SIK2 cDNA. The SIK2 mutant fragment was cloned into pCDNA3.1/Hygro^+^ (Invitrogen) vector to generate pSIK2‐mut. The small interfering (si)RNA and shRNA oligonucleotides used are listed in Table S1. pCMVTNT‐AKT was constructed by inserting the AKT gene (GenBank NM_003074.3) into the plasmid pCMVTNT. GST‐SIK2 expression vector pGEX‐SIK2 was constructed by insertion of SIK2 cDNA into pGEX‐4T‐1.

### 2.6 Cell migration and invasion assay

For the migration assay, 3 × 10^4^ cells in serum‐free media were plated into the upper chamber of Transwell insert (8‐μm pore size; BD Bioscience, San Jose, CA, USA). For the invasion assay, the Transwell insert was coated with Matrigel (BD Bioscience) and 5 × 10^4^ cells were plated onto the top of the coated insert. The medium containing 10% FBS was placed in the lower chamber as a chemoattractant. After 24 h of incubation, the cells that had not migrated or invaded through the pores in the upper chambers were removed manually with a cotton swab and then the cells on the lower surface of the filter were fixed with cool methanol for 5 min, stained with 0.1% crystal violet for 5 min, imaged (Olympus Corporation, Tokyo, Japan) and counted using imagej software (NIH, Bethesda, MD, USA).

### 2.7 Wound‐healing assay

Cells were seeded into 6‐well plates and grown to nearly 100% confluence. The same size scratch was made through the cell monolayer using a 200‐µL disposable pipette tip. After washing with PBS, fresh culture medium with 1% FBS was added and the cells were incubated at 37 °C in a 5% CO_2_ incubator. Wound closure was photographed at 0 and 48 h.

### 2.8 Co‐immunoprecipitation (Co‐IP) assay

Cells were lysed in RIPA buffer and the supernatants were collected and incubated with anti‐pan AKT antibody or anti‐SIK2 antibody or NC IgG (Table S1) overnight at 4 °C. After 2 h of incubation with agarose G plus beads, the beads were collected, washed and eluted. The eluate was separated by 12% SDS/PAGE and analyzed by Western blot analysis using anti‐SIK2 or anti‐AKT antibody.

### 2.9 Dual luciferase reporter assay

TCF‐responsive luciferase construct, Top‐Flash and its mutant Fop‐Flash (Addgene, Watertown, MA, USA) were employed to assess β‐catenin transcriptional activity. The GC cells grown in 24‐well plates for 24 h were co‐transfected with Top‐Flash and pRL‐TK (Addgene) vector or Fop‐Flash and pRL‐TK report vector. The relative luciferase activity was determined using a dual‐luciferase reporter assay kit (Promega). The pRL‐TK was used as an internal control.

### 2.10 Immunofluorescent staining

Cells were plated on a 8‐µm‐thick chip, fixed in 4% ice‐cold paraformaldehyde and permeabilized using 0.3% Triton X‐100/5% BSA/TBS for 1 h at room temperature and then incubated with antibodies against SIK2 (Abcam), β‐catenin (Abcam), LAMP1 (Cell Signaling Technology), LC3B (Abcam), PHLPP2 (Abcam) and PP2A (Cell Signaling Technology, Danvers, MA, USA) overnight at 4 °C. After three washes, cells were incubated with Alexa Fluor 488 donkey anti‐rabbit secondary antibody (Life Technologies) or Alexa Fluor 594 donkey anti‐mouse IgG (Life Technologies, Carlsbad, CA, USA). The nuclei were counterstained with DAPI (Invitrogen) and cells were visualized with a laser scanning confocal microscope.

### 2.11 Animal studies

Male nude mice (BALB/c‐null, 6 weeks old) were purchased from Shanghai Laboratory Animal Center (Chinese Academy of Sciences, Shanghai, China) and bred in special pathogen‐free conditions. All work performed with animals was approved by the Institutional Animal Care and Use Committee of Fujian Medical University. For the *in vivo* metastasis study, 3.2 × 10^6^ MGC803‐psh1SIK2 or MGC803‐pSUPER cells were resuspended in 0.1 mL serum‐free RPMI 1640 and injected into the tail veins of 10 BALB/c nude mice. At 8 weeks post injection, 10 mice in each group were euthanized and the lungs were removed and examined for evidence of metastasis using a dissecting microscopy and histopathologic analysis.

### 2.12 The Cancer Genome Atlas (TCGA) and Gene Expression Omnibus (GEO) data analysis

The mRNA‐seq data were downloaded from the TCGA stomach cancer dataset (http://tcga-data.nci.nih. gov/tcga) accessed on 28 January 2016 via RTCGAToolbox package [16]. Gene set enrichment analysis (GSEA) of mRNA‐seq data from TCGA was performed using the r package clusterProfiler. Differences were considered statistically significant at∣NES∣> 1, adjusted *P* < 0.05, and a *q*‐value of false discovery rate (FDR) < 0.25. The mRNA expression array datasets were downloaded from GEO. GSEA of mRNA array datasets was performed using the java desktop software (Oracle Corporation, Santa Clara, CA, USA. http://software.broadinstitute.org/gsea/index.jsp).

### 2.13 Statistical analysis

All data were expressed as mean ± SD. Statistical analyses were performed with prism 6 software. Statistical differences were determined by the nonparametric Mann–Whitney test or Kruskal–Wallis test, and the partial correlation analyses were based on the Pearson test. The association between SIK2 expression and the clinicopathological parameters of the GC patients were analyzed by Pearson’s chi‐square test. The survival curves were plotted using Kaplan–Meier analysis. In all cases, *P* < 0.05 was considered statistically significant.

## 3 Results

### 3.1 SIK2 is downregulated in GC and its reduced expression predicts poor prognosis of patients

**Fig 1:**
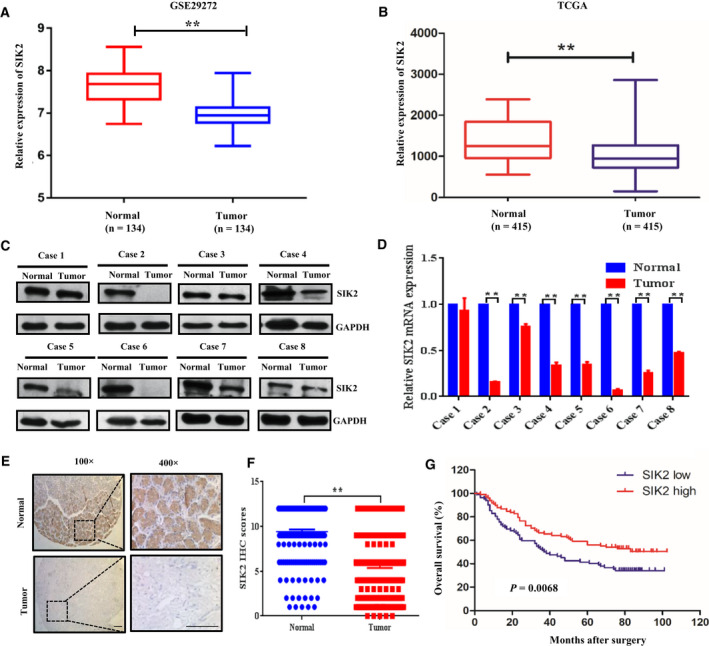
SIK2 was downregulated in GC and its reduced expression correlates with poor prognosis of patients. (A) The SIK2 expression levels of normal adjacent gastric tissues and GC tissues (*n* = 134) in the GC database of the public GEO dataset (GSE29272). (B) The SIK2 expression levels of normal adjacent gastric tissues and GC tissues (*n* = 415) GC tissues in the TCGA stomach cancer dataset. (C,D) The SIK2 expression levels in a randomly selected eight paired of GC samples and the corresponding adjacent normal tissues analyzed by immunoblotting (C) and qRT‐PCR (D). (E) Representative images of IHC staining of 180 paired GC tissues and adjacent normal tissues using an anti‐SIK2 antibody; 100× magnification, scale bar = 50 μm; 400× magnification, scale bar = 50 μm. (F) Graph showing the scores of SIK2 staining in the range of 0–12 in the GC and normal tissues of 180 patients. (G) Kaplan–Meier analysis of the correlation between the SIK2 level and the overall survival of 180 GC patients. The patients were stratified by high (IHC score > 4) versus low (IHC score < 4) expression of SIK2 (*P* = 0.0068, log‐rank test). **P < 0.01.

To assess the clinical significance of SIK2 expression, the SIK2 expression level was first analyzed using the GSE29272 data of 134 GC patients in the GEO database. Significant downregulation of SIK2 was observed in the GC tissues compared with their paired noncancerous tissues (Fig. 1A). We also examined TCGA data from 415 GC cases and found that *SIK2* expression was significantly lower in GC samples than in their adjacent normal tissues (Fig. 1B). We then examined the expression level of SIK2 in eight GC clinical specimens. As shown in Fig. 1C,D, both protein and mRNA expression levels of SIK2 were downregulated in the GC tissues compared with their adjacent normal gastric tissues. IHC was also performed to examine SIK2 protein levels in a GC tissue microarray that we prepared from 180 patients. Consistently, decreased SIK2 expression levels were seen in GC tissues compared with the paired surrounding noncancerous tissues (Fig. 1E,F). Notably, the low expression level of SIK2 was positively correlated with lymph node metastasis (*P* < 0.0001), clinical stage (*P* = 0.0025), lymphatic invasion (*P* = 0.0055) and nerve/venous invasion (*P* = 0.0291; Table 1). Accordingly, the Kaplan–Meier analysis indicated that low SIK2 expression level was significantly correlated with reduced overall survival (*P* = 0.0068, Fig. 1G). These results imply that SIK2 is frequently downregulated in GC and may function as a tumor suppressor in human GC.

**Table 1:** Association between SIK2 expression and clinicopathologic characteristics of 180 GC patients in the study cohort.

Characteristics	Cases	SIK2 expression		*P*‐value
		High	Low	
Gender				
Male	131	72	59	0.8673
Female	49	26	23	
Age (years)				
≤ 60	74	37	37	0.3625
> 60	106	61	45	
Tumor size (cm)				
≤ 5	108	56	52	0.4460
> 5	72	42	30	
Histology differentiation				
Poor	102	53	49	0.4549
Well or moderate	78	45	33	
Depth of tumor invasion				
T1	5	4	1	0.6704
T2	18	9	9	
T3	41	23	18	
T4	116	62	54	
Lymphatic metastasis				
N0	44	32	12	<0.0001*
N1	73	46	27	
N2	40	12	28	
N3	23	8	15	
Clinical stage				
Ⅰ	24	19	5	0.0025*
Ⅱ	52	33	19	
Ⅲ	104	46	58	
Lymphatic invasion				
Absent	44	32	12	0.0055*
Present	136	66	70	
Nerve/Venous invasion				
Absent	116	56	60	0.0291*
Present	64	42	22	

* Indicates statistical significance.

### 3.2 SIK2 inhibits the malignant phenotype of GC cells both *in vitro* and *in vivo*

**Fig 2:**
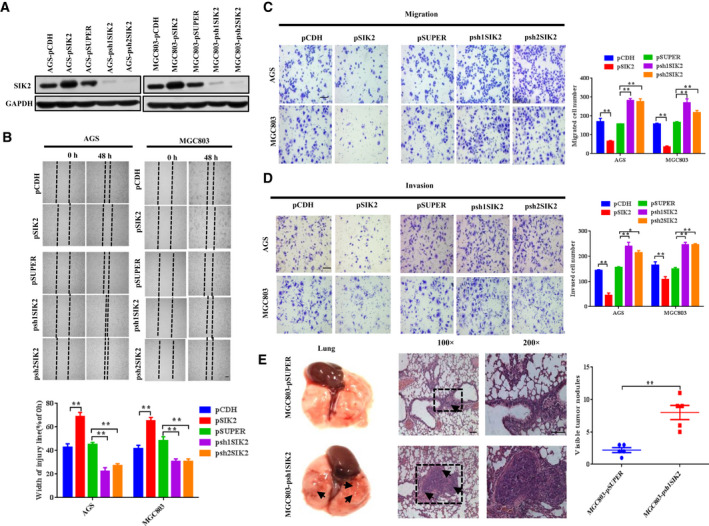
SIK2 inhibits the malignant phenotype of GC cells both *in* *vitro* and *in vivo*. (A) Western blot analysis confirming stable overexpression or knockdown of SIK2 in AGS and MGC803 cells. Glyceraldehyde‐3‐phosphate dehydrogenase (GAPDH) was used as loading control. (B) Relative motility as determined by the ability of the GC cells to close a wound made by creating a scratch through a lawn of confluent cells. (C) Relative migration of the GC cells through an uncoated filter toward serum‐containing medium in a Boyden chamber assay. Scale bar = 200 μm. (D) Relative invasion of the GC cells through a layer of Matrigel coated on the filter of a Boyden chamber. Scale bar = 200 μm. (E) Representative gross appearance of the lungs (left); H&E staining of lung metastases (middle) and number of visible surface metastatic lesions in mice (right) 8 weeks after tail vein injection of 3.2 × 10^6^ MGC803‐pSUPER or MGC803‐psh1SIK2 cells. *n* = 10 per group. Scale bar = 50 μm. Data are expressed as mean ± SD from at least three independent experiments with triple replicates per experiment. *P < 0.05, **P < 0.01.

Given that SIK2 was downregulated in GC and its downregulation was correlated with lymph node metastasis and shorter survival, it is reasonable to postulate that loss of SIK2 augments GC cell aggressive behavior. To test this hypothesis, we first prepared GC sublines in which *SIK2* was constitutively knocked down or overexpressed (Fig. 2A) and evaluated the effect of SIK2 on the malignant phenotype of these GC cells. As shown in Figs 2B‐D, knocking down endogenous SIK2 in MGC803 and AGS cells substantially increased the cell migration and invasion as determined by wound‐healing/scratch assay (Fig. 2B) and Boyden two‐chamber assay (Fig. 2C,D), whereas overexpression of SIK2 significantly reduced the cell migratory and invasive capacity. The effect of SIK2 knockdown on metastatic potential *in vivo* was examined by injecting SIK2‐knockdown MGC803‐psh1SIK2 cells or MGC803‐pSUPER control cells into the caudal vein of BALB/c nude mice. The appearance of metastases to the lung was examined 8 weeks after the tail vein injection. By gross appearance, mice injected with MGC803‐psh1SIK2 cells generated a significantly larger number of nodules in their lungs than did those injected with the control cells (Fig. 2E). Hematoxylin and eosin (H&E) staining confirmed that knockdown of SIK2 in the GC cells remarkably increased the formation of metastases to the lung compared with the control cells. Together, these results indicate that SIK2 may function to suppress GC cell migration, invasion and metastasis.

### 3.3 SIK2 suppresses epithelial‐mesenchymal transition (EMT) via regulation ofβ‐catenin signaling

**Fig 3:**
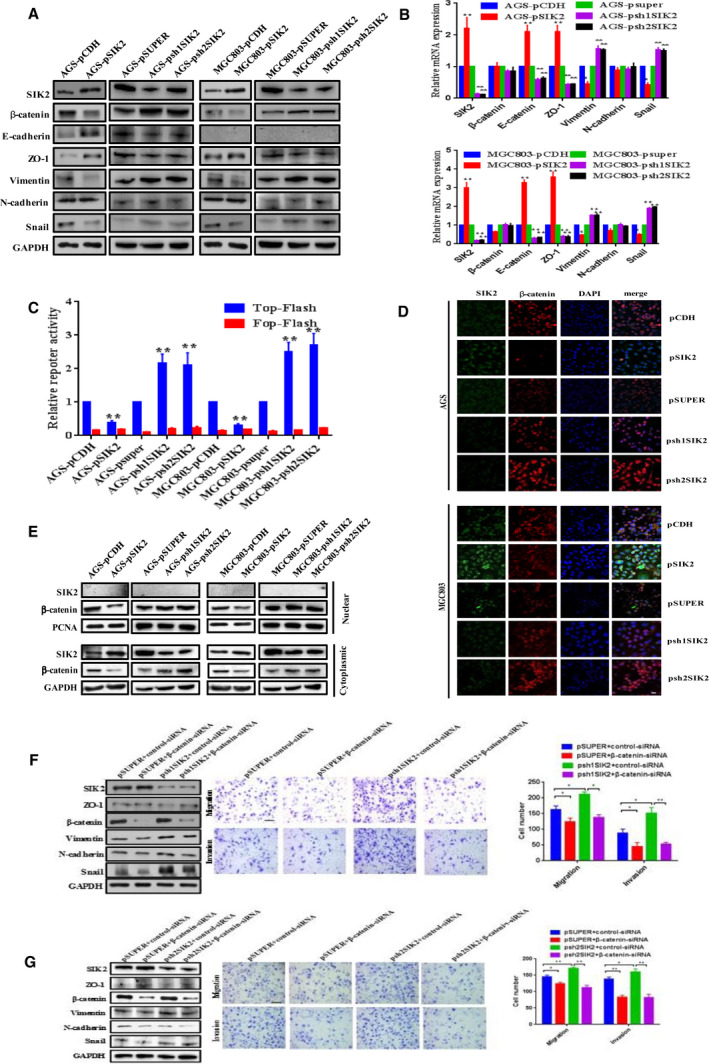
SIK2 suppresses EMT via regulation of β‐catenin signaling. (A) Western blot analysis of the expression of EMT markers in the indicated cells. (B) qRT‐PCR analysis of the expression of EMT markers in the indicated cells. (C) TOP or FOP Flash reporter assay to evaluate β‐catenin transcriptional activity in the indicated cells. The detected reporter activity was normalized to Renilla activity. (D) Immunofluorescence staining of nuclear and cytoplasmic β‐catenin in the indicated cells. Expression of SIK2 was stained in green and β‐catenin in red. DAPI was used as a nuclear counterstain. Scale bar = 25 µm. (E) Western blot analysis of the levels of nuclear and cytoplasmic β‐catenin in the indicated cells. PCNA and GAPDH served as nuclear and cytoplasmic protein loading controls, respectively. (F,G) Effect of siRNA‐mediated knockdown of β‐catenin in the SIK2‐knockdown MGC803‐psh1SIK2 (F) and MGC803‐psh2SIK2 (G) cells, respectively, on the expression of EMT markers, and the cell migration and invasion. Scale bar = 200 µm. Data were expressed as mean ± SD from at least three independent experiments with triple replicates per experiment. *P < 0.05; **P < 0.01.

Given the observation that loss of SIK2 caused GC cells to act more aggressively, we suspected that these changes might result from the ability of SIK2 to suppress EMT, an important step for cancer cell metastasis. To probe the possibility of EMT to account for the invasiveness of the SIK2‐deficient GC cells, we examined the expression of markers associated with EMT by Western blot analysis and real‐time PCR in the control and SIK2‐knockdown cells using two specific shRNAi, targeting different regions of *SIK2* messenger (m)RNA. The results showed that knockdown of *SIK2* in AGS and MGC803 cells upregulated the expression of EMT‐related markers such as Vimentin, Snail and β‐catenin, but reduced the expression level of epithelial marker ZO‐1 (Fig. 3A). In contrast, overexpression of SIK2 produced the opposite effect. Of note, no significant change in the expression level of N‐cadherin was detected across any of the cell lines and E‐cadherin was not detectable in any of the MGC803‐derived cell lines. Similar results regarding the pattern of altered expression of EMT‐related markers were obtained with quantitative real‐time polymerase chain reaction (qRT‐PCR) analysis (Fig. 3B).

Several signaling pathways are known to play important roles in EMT, such as TGF‐β, Notch, JAK/STAT3 and Wnt/β‐catenin [17]. Aberrant β‐catenin signaling has been shown to be an essential regulator of EMT in many types of malignancies, e.g. GC [18, 19]. Having demonstrated that SIK2 markedly affected the expression level of β‐catenin (Fig. 3A), we went further to investigate whether SIK2 could affect β‐catenin transcriptional activation activity using Top/Fop Flash reporter assays. Figure 3C showed that overexpression of SIK2 in MGC803 and AGS cells significantly decreased the β‐catenin transcriptional activity, whereas knockdown of SIK2 substantially increased the transcriptional activity of β‐catenin. Fluorescent microscopy was then utilized to detect β‐catenin intracellular localization. The results showed that SIK2 knockdown in GC cells markedly enhanced cytoplasmic and nuclear accumulation of β‐catenin, which was in sharp contrast to the marked reduction of β‐catenin accumulation when SIK2 was overexpressed (Fig. 3D). Consistent with the results obtained with fluorescent microscopy, fractionation experiments also demonstrated that the level of β‐catenin in cytoplasmic and nuclear extracts was increased or reduced accordingly in response to knockdown or overexpression of SIK2 (Fig. 3E). To verify further the important role of β‐catenin in the context of the SIK2‐regulated EMT process, β‐catenin was knocked down with a specific siRNA, targeting β‐catenin in the MGC803‐psh1SIK2 cells; the expression pattern changes of the EMT‐associated markers were then examined. As shown in Fig. 3F,G, knockdown of β‐catenin effectively reversed the effect of SIK2 loss on the expression of EMT markers, and consequent securing of epithelial traits translated into less aggressive phenotypes, as reflected by diminished cell migration and invasion. These results suggest that it is the activation of β‐catenin that accompanies downregulation of SIK2 expression driving the EMT process in GC cells.

### 3.4 SIK2 inhibits AKT/GSK3β/β‐catenin signaling

**Fig 4:**
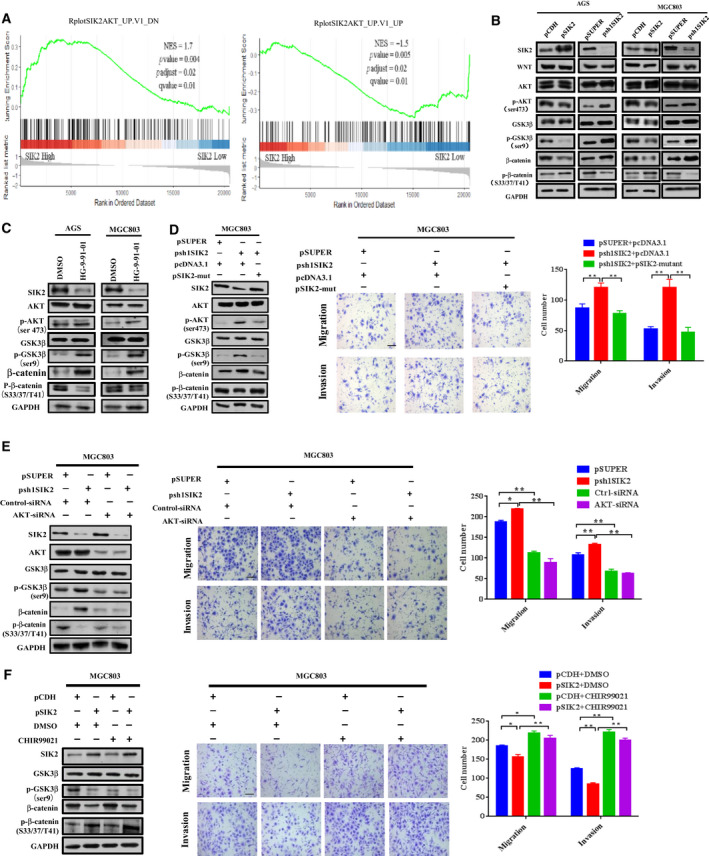
SIK2 inhibits AKT/GSK3β/β‐catenin signaling. (A) Analysis of the AKT signal enrichment scores between SIK2 high and low expression groups according to the TCGA stomach cancer dataset by GSEA. (B) Western blot analysis of the expression of AKT/GSK3β/β‐catenin signaling molecules in the SIK2‐overexpressed or ‐knockdown AGS and MGC803 cells. (C) Effect of addition of SIK2 inhibitor HG‐9‐91‐01 (15 μm, 24 h) on the expression of AKT/GSK3β/β‐catenin signaling molecules in AGS and MGC803 cells. (D) Effect of reintroduction of a siRNA‐resistant SIK2 construct into the SIK2‐knockdown MGC830 cells on the expression of AKT/GSK3β/β‐catenin signaling molecules (left) and the cell migratory and invasive abilities (middle and right). Scale bar = 200 µm. (E) Effect of AKT knockdown in SIK2‐knockdown MGC830 cells on the expression of AKT/GSK3β/β‐catenin signaling molecules (left) and the cell migratory and invasive ability (middle and right). Scale bar = 200 µm. (F) Effect of addition of GSK3β inhibitor CHIR99021 (3 μm, 24 h) in SIK2‐overexpressed MGC830 cells on the expression of AKT/GSK3β/β‐catenin signaling molecules (left) and the cell migratory and invasive abilities (middle and right). Scale bar = 200 µm. Data were expressed as mean ± SD from at least three independent experiments with triple replicates per experiment. *P < 0.05; **P < 0.01.

PI3K regulatory subunit p85α is known to be a direct catalytic target of SIK2 whose activation could increase the activity of the PI3K/AKT pathway [20]. Phosphorylation of GSK3β at Ser 9 by AKT, which deactivates GSK3β, allows β‐catenin to accumulate in the cytoplasm and then translocate into the nucleus and elicit transcriptional events leading to more malignant behaviors [21]. Therefore, we were interested in examining whether the AKT/GSK3β pathway participated in the regulation of β‐catenin mediated by the loss of SIK2. To evaluate the relationship between SIK2 and AKT expression levels, mRNA‐seq data in GC (*n* = 415) were downloaded from the TCGA database and GSEA analysis was performed. The results showed that SIK2 expression was positively correlated with the AKT_UP.V1_DN gene set (NES = 1.7, *P* = 0.004, *P*_adjust_ = 0.02, FDR *q* = 0.01) and negatively correlated with the AKT_UP.V1_UP gene set (NES = −1.5, *P* = 0.005, *P*_adjust_ = 0.02, FDR *q* = 0.01; Fig. 4A). Being assured of this negative correlation between SIK2 expression and AKT signal enrichment, we proceeded to examine the expression levels of both total and phosphorylated AKT, GSK3β and β‐catenin in the AGS and MGC803 cells with SIK2 overexpressed or knocked down. As expected, knockdown of SIK2 substantially increased phosphorylation of AKT at Ser473 and GSK3β at Ser9, which subsequently increased total β‐catenin levels due to reduced phosphorylation of β‐catenin at Ser33/37/Thr41 being targeted for degradation (Fig. 4B). In contrast, overexpression of SIK2 mostly produced the opposite effects. In addition, treatment of the parental AGS and MGC803 with pan‐SIK inhibitor HG‐9‐91‐01 [22] recapitulated the results obtained with shRNAi‐mediated knockdown of SIK2 (Fig. 4C). To further determine whether the observed activation of AKT/GSK3β/β‐catenin signaling was specific to the loss of SIK2, we re‐introduced SIK2 in the SIK2‐knockdown MGC803 cells with the sh1SIK2‐resistant SIK2 construct pSIK2‐mut. Rescue of SIK2 expression by pSIK2‐mut completely reversed the effect of SIK2 knockdown on AKT/GSK3β/β‐catenin signaling, in line with the impaired cell ability to migrate and invade (Fig. 4D). The assumption that AKT/GSK3β signaling contributes to regulation of β‐catenin in the contextual loss of SIK2 was further supported by the observation that knockdown of AKT in the MGC803‐psh1SIK2 cells reduced GSK3β phosphorylation at Ser9, thus decreasing the β‐catenin level, which accordingly impaired the cell migratory and invasive capability (Fig. 4E). Conversely, treatment of the SIK2‐overexpressing MGC803‐pSIK2 cells with GSK3β inhibitor CHIR99021, which impairs the ability of GSK3β to phosphorylate β‐catenin for degradation [23] partially restored total β‐catenin levels initially inhibited by SIK2 overexpression, resulting in a corresponding increase of cell migration and invasion (Fig. 4F). Collectively, these results suggest that the inhibitory effect of SIK2 on GC cell migration and invasion is executed via suppression of AKT/GSK3β/β‐catenin signaling.

### 3.5 SIK2 inactivates AKT by upregulation of protein phosphatases PHLPP2 and PP2A

**Fig 5:**
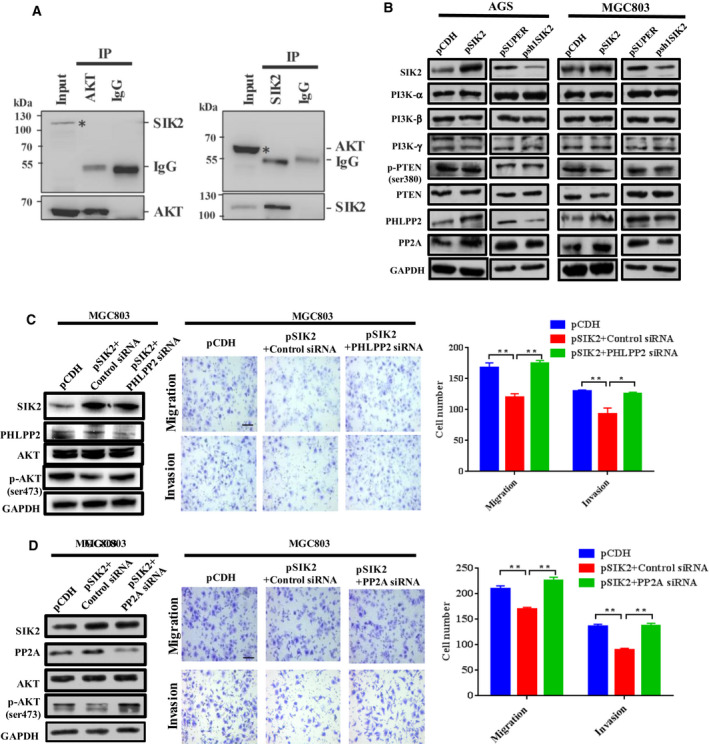
SIK2 inactivates AKT by upregulation of protein phosphatases PHLPP2 and PP2A. (A) The immunoprecipitate by anti‐AKT (pan) antibodies was examined for the presence of SIK2 or vice versa in MGC803 cells assessed by Western blot analysis. The input sample (*) served as the positive control and IgG sample was the NC. The actual presence of AKT or SIK2 protein *per se* in the immunocomplex precipitated by anti‐AKT antibody or anti‐SIK2 antibody is shown in the bottom panels. (B) Western blot analysis of the expression of PI3K subunits, phospho‐PTEN, PTEN, PHLPP2 and PP2A in the SIK2‐overexpressed or ‐knockdown AGS and MGC803 cells. (C,D) Effect of siRNA‐mediated knockdown of PHLPP2 (C) or PP2A (D) in the SIK2‐overexpressed MGC803 cells on the expression of total and phosphorylated AKT, and the cell migratory and invasive abilities. Scale bar = 200 µm. Data are expressed as mean ± SD from at least three independent experiments with triple replicates per experiment. *P < 0.05; **P < 0.01.

A wide variety of cellular processes could be regulated by the reversible phosphorylation of proteins balanced by protein kinases and protein phosphatases. In the case of AKT, its activation is accomplished by two successive phosphorylations: first by PDK1 on the activation loop (Thr308), then followed by phosphorylation on the hydrophobic motif (Ser473) [24]. Its inactivation is mediated by two mechanisms: dephosphorylation of PIP3 to remove the activating lipid second messenger by lipid phosphatase PTEN, and direct dephosphorylation of AKT by protein phosphatase 2A (PP2A) at Thr308 and by the PH domain leucine‐rich repeat protein phosphatase (PHLPP) at Ser473 [25]. Since SIK2 itself is a kinase and loss of SIK2 has been shown to decrease the level of AKT phosphorylation [20], we were curious as to whether there was an interaction between endogenous SIK2 and AKT in GC cells. A reciprocal Co‐IP study showed that the precipitated proteins by anti‐pan‐AKT antibody did not contain SIK2 (Fig. 5A, left panel). Likewise, in the reverse immunoprecipitation study, AKT was not detectable in the immunocomplex precipitated by anti‐SIK2 antibody. These data indicate that SIK2 had no physical contact with AKT. Intriguingly, although SIK2 had no effect on the expression of PI3Kα, PI3Kβ, PI3Kγ, p‐PTEN or PTEN, overexpression or knockdown of SIK2 respectively increased or decreased the protein levels of PHLPP2 and PP2A in AGS and MGC803 cells (Fig. 5B). To determine whether SIK2‐mediated AKT inactivation was attributable to dephosphorylation of AKT by PHLPP2 and PP2A, we transiently knocked down PHLPP2 or PP2A in the MGC803‐pSIK2 cells and checked for phosphorylation status of AKT. As expected, knockdown of either PHLPP2 or PP2A significantly increased the suppressed pAKT level that accompanied SIK2 overexpression, which also restored the cell migratory and invasive ability (Fig. 5C). To provide further assurance that AKT signaling was affected by the two protein phosphatases, we transiently knocked down PHLPP2 or PP2A using two specific siRNA in both AGS and MGC803 cells and then examined the levels of AKT phosphorylation. The results showed that the siRNA‐mediated knockdown of either PHLPP2 or PP2A in the GC cells substantially increased AKT phosphorylation (Fig. S1), suggesting that AKT is indeed the target of PHLPP2 or PP2A for the dephosphorylation. These data indicate that SIK2‐mediated inactivation of AKT acts, at least in part, through upregulation of PHLPP2 and PP2A.

### 3.6 SIK2 inhibits mTORC1‐dependent autophagic degradation of PHLPP2 and PP2A

**Fig 6:**
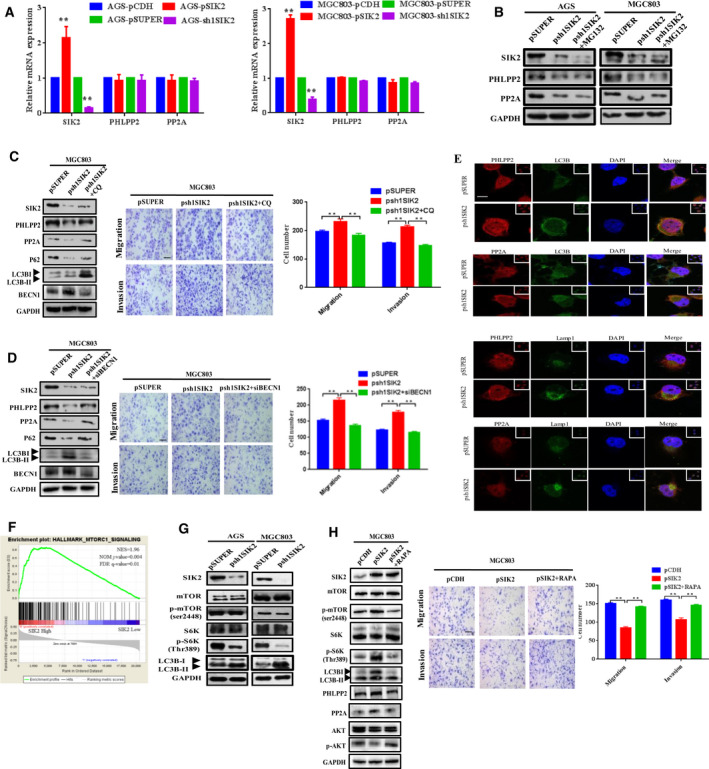
SIK2 inhibits mTORC1‐dependent autophagic degradation of PHLPP2 and PP2A. (A) The mRNA expression levels of PHLPP2 and PP2A in the SIK2‐overexpressed or ‐knockdown AGS and MGC803 cells assessed by qRT‐PCR. (B) Effect of proteasome inhibitor MG132 (5 μm, 24 h) on the expression of PHLPP2 and PP2A in the SIK2‐knockdown AGS‐psh1SIK2 and MGC803‐psh1SIK2 cells. (C,D) Effect of the autophagy inhibitor CQ (C) or knockdown of Beclin‐1 (BECN1) (D) on the expression of PHLPP2, PP2A and autophagic markers in the SIK2‐knockdown MGC803‐psh1SIK2 cells, and the cell migratory and invasive abilities. Scale bar = 200 µm. (E) Immunostaining analysis of colocalization of PHLPP2 or PP2A with LC3B and LAMP1 in MGC803‐psh1SIK2 and the control cells. Cells were fixed and labeled with anti‐PHLPP2 or anti‐PP2A (green) and anti‐LC3B or anti‐LAMP1 (red) antibodies. Yellow = merge/colocalization. White boxes in the images are pictures of the area of the cells taken with low magnification. Scale bar = 10 µm. (F) Analysis of the mTORC1 signal enrichment scores between SIK2 high and low expression groups according to the TCGA stomach cancer dataset using the GSEA program. (G) Western blot analysis of the expression of autophagy signaling molecules in the SIK2‐knockdown AGS‐psh1SIK2 and MGC803‐psh1SIK2 cells. (H) Effect of addition of mTOR inhibitor rapamycin (100 ng·mL^−1^, 6 h) in the SIK2‐knockdown MGC830 cells on the expression of autophagy markers and AKT phosphorylation as well as the cell migratory and invasive abilities. Scale bar = 200 µm. Data are expressed as mean ± SD from at least three independent experiments with triple replicates per experiment. **P < 0.01.

**Fig 7:**
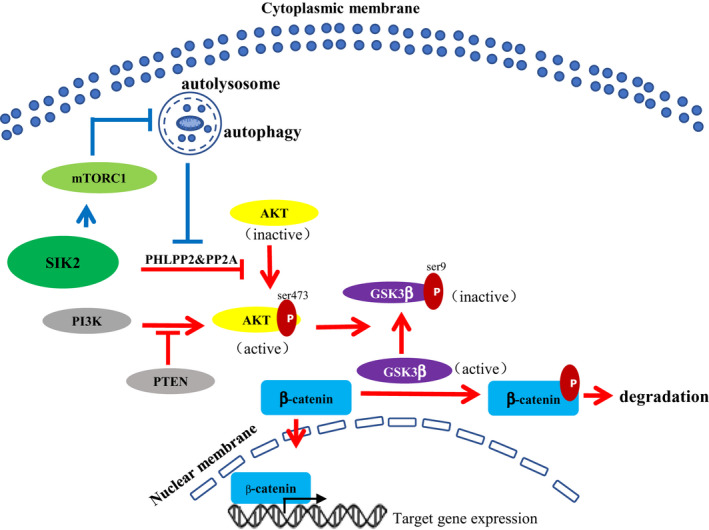
Schematic models for the mechanism by which SIK2 functions to suppress the malignant phenotypes of GC by activation of mTORC1 to inhibit autophagic degradation of protein phosphatases PP2A and PHLPP2, thus increasing dephosphorylation and inactivation of AKT/GSK3β/β‐catenin signaling.

SIK2 has been shown to be indispensable for autophagosome processing [10] and mTORC1 is known to have a direct interaction with and to inhibit the ULK1 complex, an essential component in autophagy initiation [26]. Given that SIK2 was able to upregulate the expression of PHLPP2 and PP2A, we hypothesized that SIK2 might phosphorylate and activate mTORC1 to inhibit autophagic degradation of PHLPP2 and PP2A. To exclude the possibility that SIK2 may transcriptionally regulate PHLPP2 and PP2A expression, we performed qRT‐PCR to measure mRNA expression levels of *PHLPP2* and *PP2A* in the SIK2‐overexpressed or ‐knockdown GC cells. As shown in Fig. 6A, neither overexpression or knockdown of SIK affected the transcript levels of *PHLPP2* and *PP2A*, suggestive of a possible posttranscriptional mechanism that may involve in SIK2‐induced upregulation of the protein levels of these two phosphatases. To examine whether SIK2 stabilizing effect on PHLPP2 and PP2A acted through proteasome pathway, the AGS‐psh1SIK2 and MGC803‐psh1SIK2 cells were treated with the proteasome inhibitor MG132. As shown in Fig. 6B, MG132 treatment failed to rescue the reduction of PHLPP2 and PP2A expression resulting from SIK2 knockdown, indicating that the degradation of PHLPP2 and PP2A was probably proteasome‐independent. Instead, the autophagy inhibitor chloroquine (CQ), whose effectiveness was confirmed by its ability to decrease the level of autophagic existence markers LC3‐II/I and Beclin‐1 but increase the level of autophagic defect marker p62, restore PHLPP2 and PP2A protein levels initially suppressed by SIK2 knockdown, and reduce the cell migratory and invasive capability (Fig. 6C). A similar effect was obtained using a genetic approach to knock down the core autophagy gene *Beclin‐1* (Fig. 6D). To further confirm that it was the autophagic flux that contributed to an increase of lysosomal degradation of PHLPP2 and PP2A when SIK2 was lost, the presence or transition of these two protein phosphatases within autophagosomes and lysosomes was assessed by immunofluorescent staining (Fig. 6E). Results showed that both PHLPP2 and PP2A were colocalized with puncta positive for LC3B and Lamp1 more prominently in the SIK2 knockdown cells than in the control cells, suggesting that loss of SIK2 activated the autophagy signaling pathway, leading to autophagosome‐mediated and eventually lysosomal degradation of PHLPP2 and PP2A. Given the involvement of SIK2 in autophagy‐related regulation of PHLPP2 and PP2A, we then tested whether SIK2‐induced inhibition of PHLPP2 and PP2A autophagic degradation was mediated through the signaling pathway of mTORC1, a well‐known major suppressor of autophagy induction [27]. Using the independent GC gene expression datasets (GSE15459) from public database GEO, we found that SIK2 was positively correlated with mTORC1 enrichment scores (Fig. 6F). Such positive correlation was further reflected in SIK2‐knockdown GC cells, where phosphorylation of mTORC1 at Ser2448 and its downstream substrate RPS6KB1/S6K1 at Thr389 was significantly diminished with concurrent increase of the level of LC3‐II/I (Fig. 6G). Confirmed another way around, treatment of SIK2‐overexpressed MGC803‐pSIK2 cells with mTORC1 inhibitor rapamycin (RAPA) markedly attenuated SIK2‐induced activation of mTORC1 and concomitantly increased the LC3‐II/I level, which decreased the expression levels of PHLPP2 and PP2A but increased AKT phosphorylation, ultimately leading to increased migration and invasion of the cells (Fig. 6H). Our data are most consistent with the concept, as illustrated in Fig. 7, that SIK2 functions to suppress the malignant phenotypes of GC by inhibition of autophagy‐mediated degradation of protein phosphatases PP2A and PHLPP2 via mTORC1 signaling, thus inactivating AKT/GSK3β/β‐catenin signaling primarily owing to increased dephosphorylation of AKT.

## 4 Discussion

Increasing evidence suggests that SIK2 modulates a wide variety of biological functions and acts as a signal transmitter in several pathways important to tumorigenesis and progression of cancer cells and can execute pro‐ or anti‐tumorigenic effects dependent on cell type or context [8]. Although SIK2 presents as a oncogenic marker on most occasions, a few studies have also revealed it to be a tumor suppressor [14, 28, 29]. In this study, we demonstrated that downregulation of SIK2 was frequently observed in GC tissues as compared with their adjacent normal gastric tissues, and was closely correlated with poor clinical outcome. Knockdown of SIK2 in GC cells promoted cell migration and invasion *in* *vitro* as well as metastatic potential *in vivo*. Overexpression of SIK2 in GC cells inhibited EMT process by decreasing the intracellular β‐catenin level due to attenuation of AKT/GSK3β signaling, resulting primarily from increased dephosphorylation of AKT by the protein phosphatases PHLPP2 and PP2A. These results suggest that SIK2 functions as a tumor suppressor in GC and unveils a novel mechanism of SIK2 in GC development and progression.

The metastasis of cancer cells accounts for more than 90% of cancer‐related mortality [30] and EMT has been considered an important step in cancer cell metastasis [31]. During the EMT process, epithelial cells lose polarity and gain invasive properties to become mesenchymal cells [32]. Until recently, there is only one report that focused on the role of SIK2 in EMT and tumor metastasis; that report demonstrated an inhibitory effect of SIK2 on migration and invasion of breast cancer cells by blocking EMT with simultaneous blockage of Ras/ERK and PI3K/AKT pathways [14]. In our *in* *vitro* gain and loss of function study, we found that knockdown of SIK2 in GC cells significantly increased expression of EMT markers such as Vimentin and Snail but lost expression of epithelial marker ZO‐1. Those SIK2 knockdown cells that have gone through an EMT displayed more aggressive phenotypes, such as enhanced migration and invasion *in* *vitro* and metastatic potential *in vivo*. In contrast, the opposite effect was seen with ectopic expression of SIK2. Thus, we assume that SIK2 may function as a metastasis suppressor in GC by inhibiting the EMT process.

Although the permissive signal regulating the EMT program in GC remains largely unclear, we have previously demonstrated that Wnt/β‐catenin signaling is essential for GC cells to undergo the EMT process [19, 33]. Activation of Wnt signaling would stabilize β‐catenin, resulting in its accumulation in the cytoplasm and subsequently in the nucleus, where it ties in with T cell factor/lymphoid enhancer‐binding factor (TCF/LEF) to facilitate transcription of various target genes, including those related to EMT [21, 34, 35]. In the current study, we showed that SIK2 overexpression significantly reduced the intracellular levels of β‐catenin, whereas its knockdown increased β‐catenin expression. Such SIK2‐dependent alteration of β‐catenin level was accompanied by the change in GC cell aggressive phenotype. Moreover, the observation that siRNA knockdown of *β‐catenin* in MGC803‐psh1SIK2 cells reversed EMT and reduced the cell migratory and invasive ability, further supports the idea that SIK2 deficiency‐induced EMT and aggressiveness was executed through β‐catenin.

We then sought to uncover the signaling mechanism by which SIK2 regulates β‐catenin in GC cells. It is well established that the cellular localization and accumulation of β‐catenin are tightly regulated by GSK3β, a direct target of AKT, for phosphorylation, ubiquitination and proteasomal degradation of β‐catenin [36, 37, 38]. Bioinformatic analysis using the GSEA program and our experimental result showed that SIK2 expression was inversely correlated to the level of p‐AKT (Ser473) in GC. In addition, knockdown of *SIK2* in GC cells caused an increase in Ser9 phosphorylation of GSK‐3β known to be inactive in mediating the degradation of β‐catenin. We also found that treatment with a specific AKT siRNA in SIK2 knockdown cells reduced GSK3β phosphorylation at Ser9, which was accompanied by a decrease in the level of β‐catenin. Although it is not clear whether the increase in β‐catenin is the sole factor that determines the invasive phenotype due to loss of SIK2 or whether there are additional pathways involved, the results of this study implicate SIK2 as mediating β‐catenin expression and function via the AKT/GSK‐3β signaling pathway in GC cells.

Exquisite control of the balance between protein phosphorylation and protein dephosphorylation, catalyzed respectively by protein kinases and protein phosphatases, is indispensable for cellular homeostasis. Deregulation of this balance would result in various pathogenic states and is particularly prevalent in cancer owing to aberrant phosphorylation and dephosphorylation of signaling molecules that control cell growth and survival [39]. For example, the lipid phosphatase PTEN, one of the most investigated tumor suppressors, can dephosphorylate PIP3, thus abrogating the oncogenic kinase AKT activation. The revelation of a group of protein phosphatases that could legitimately dephosphorylate and inactivate AKT, presents another negative controller of the PI3K oncogenic pathway [40]. SIK2 itself is a protein kinase; however, controversy exists regarding its effect on AKT phosphorylation and signaling. Miranda et al. reported that activated SIK2 in ovarian cancer cells triggered phosphorylation of p85α, the regulatory subunit of the PI3K complex, and concomitant phosphorylation of AKT [20]. However, another study in breast cancer cells found that SIK2 expression reduced AKT phosphorylation levels, whereas depletion of SIK2 led to a substantial increase in p‐AKT levels [14]. Coincident with the latter study, we have now shown that overexpression or knockdown of SIK in GC cells diminished or elevated phosphorylation of AKT at Ser47. In pursuit of the mechanism by which SIK2 modulates AKT phosphorylation, we found that SIK2 did not interact directly with AKT, as shown in a reciprocal Co‐IP study, nor did SIK2 affect the expression of PI3Kα, PI3Kβ, PI3Kγ, p‐PTEN or PTEN. Intriguingly, we observed a significant increase of PHLPP2 and PP2A protein levels in the SIK2‐overexpressing GC cells. That reduction of AKT phosphorylation by SIK2 is mediated by PHLPP2 or PP2A was further supported by the evidence that knockdown of PHLPP2 or PP2A in SIK2‐overexpressed cells restored the p‐AKT level.

SIK2 has been shown to be a critical determinant in autophagy progression [10, 41] and mTORC1 plays a vital role in autophagy induction [26]. This led us to hypothesize that SIK2 might activate mTORC1 to inhibit autophagic degradation of PHLPP2 and PP2A. We found that mRNA levels of PHLPP2 and PP2A were not significantly altered in SIK2‐overexpressed and ‐knockdown cells, strengthening the concept that SIK2 affects PHLPP2 and PP2A expression at the posttranscriptional or posttranslational level, or both. Although protein phosphatases could be degraded via the ubiquitin‐proteasome pathway [42], our observation that treatment with proteasome inhibitor could not restore PHLPP2 and PP2A expression initially reduced by SIK2 knockdown suggests that the degradation of PHLPP2 and PP2A was likely to be proteasome‐independent. Instead, our further investigations disclosed that SIK2 augmented phosphorylation and activation of mTORC1 that, as expected, inhibited autophagic degradation of PHLPP2 and PP2A. It may be noteworthy that autophagy was only recently linked to EMT. Recent studies indicate that these two processes may be interconnected in a complex relationship [43, 44]. Cells that have gone through EMT require autophagy activation for survival during the metastatic spreading. Conversely, autophagy could function as a onco‐suppressive player to restrain the early stages of metastasis by specifically destabilizing crucial mediators of EMT. Currently, there is very limited information available with respect to the molecular hubs at the interplay between autophagy and EMT. In the context of SIK2, we found that loss of the SIK2‐activated autophagy signaling pathway led to an increase of autophagy‐lysosomal degradation of PHLPP2 and PP2A, and to more aggressive phenotypes of SIK2‐deficient cells. Although in this study SIK2 is found to contribute to cellular autophagy and EMT in GC, detailed molecular mechanisms by which they functionally interact with each other cooperatively to regulate downstream signaling for cancer progression remain to be fully elucidated.

## 5 Conclusions

In summary, the present work is the first demonstration of the prognostic value of SIK2 for patients with GC and of the suppression of GC tumorigenesis by SIK2 and progression by activation of mTORC1 to inhibit autophagic degradation of protein phosphatases PP2A and PHLPP2, thus increasing dephosphorylation and inactivation of AKT/GSK3β/β‐catenin signaling. Although it should be recognized that SIK2 inhibitors have preclinically proven effective against several SIK2‐proficient cancers, a therapeutic approach directed at enhancing the level or functional activity of SIK2 may represent another important strategy for treatment of SIK2‐deficient cancer types such as GC, where SIK2 serves as a tumor suppressor.

## Conflict of interest

The authors declare no conflict of interest.

## Author contributions

XJL and XL conceived and designed the study. XMD, YHZ, XHL, XXH, YZ and CRX developed the methodology. XMD, YHZ, XHL, XXH, YZ and CRX were involved in acquisition of data (acquired and managed patients, provided facilities, etc.). XMD, YHZ, WNC, XJL and XL were involved in analysis and interpretation of data (e.g. statistical analysis, biostatistics, computational analysis). XJL and XL wrote, reviewed and revised the manuscript. YHZ and XXH gave administrative, technical and material support. XJL and XL supervised the study.

## Supporting information


**Fig. S1.** Western blot analysis of expression of p‐AKT and total AKT in the AGS and MGC803 cells with either PHLPP2 or PP2A knockdown using two specific siRNA.

**Table S1.** Antibodies used in the study.
**Table S2.** Oligonucleotides used for cloning and qRT‐PCR.
