# Supplementary material for: SIK2 represses AKT/GSK3β/β‐catenin signaling and suppresses gastric cancer by inhibiting autophagic degradation of protein phosphatases
Source: Mol Oncol. 2020 Nov 20;15(1):228–45. doi: 10.1002/1878-0261.12838 (PMC7782074; doi:10.1002/1878-0261.12838)
Supplement: Supplementary file 1 — Fig. S1. Western blot analysis of expression of p‐AKT and total AKT in the AGS and MGC803 cells with either PHLPP2 or PP2A knockdown using two specific siRNA. [file MOL2-15-228-s001.pdf]

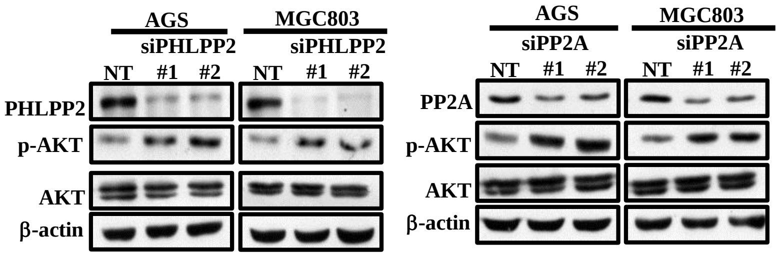

Supplementary Fig. S1. Western blot analysis of expression of p-AKT and total AKT in the AGS and MGC803 cells with either PHLPP2 or PP2A knockdown using two specific siRNAs.
