# Supplementary material for: SIK2 represses AKT/GSK3β/β‐catenin signaling and suppresses gastric cancer by inhibiting autophagic degradation of protein phosphatases
Source: Mol Oncol. 2020 Nov 20;15(1):228–45. doi: 10.1002/1878-0261.12838 (PMC7782074; doi:10.1002/1878-0261.12838)
Supplement: Supplementary file 2 — Table S1. Antibodies used in the study. Table S2. Oligonucleotides used for cloning and qRT‐PCR. [file MOL2-15-228-s002.docx]

**Supplementary Table S1**. Antibodies used in the study.

| **Antibodies** | **Manufacturer** | **Catalogue No.** | **Dilution** |
| --- | --- | --- | --- |
| Anti-PHLPP2 | Abcam | ab71973 | 1:1000 for WB; 1:200 for IF |
| Anti-SIK2 | Abcam | ab115567 | 1:50 for IHC/IF |
| Anti-β-catenin | Abcam | ab6302 | 1:200 for IF |
| Anti-β-catenin | CST | #8480 | 1:1000 for WB |
| Anti-p-β-catenin | CST | #9561 | 1:1000 for WB |
| Anti-E-cadherin | Abcam | ab1416 | 1:1000 for WB |
| Anti-N-cadherin | Abcam | ab76057 | 1:1000 for WB |
| Anti-Vimentin | Abcam | ab8978 | 1:1000 for WB |
| Anti-actin | Abcam | ab8226 | 1:5000 for WB |
| Anti-GAPDH | Abcam | ab127428 | 1:5000 for WB |
| Anti-AKT (pan) | CST | #4685 | 1:1000 for WB  1:100 for Co-IP |
| Anti-IgG | CST | #2729 | 2ul for Co-IP |
| Anti-pAKT (Ser473) | CST | #4060 | 1:1000 for WB |
| Anti-SIK2 | CST | #6919 | 1:1000 for WB |
| Anti-PP2A | CST | #2038 | 1:1000 for WB  1:200 for IF |
| Anti-Wnt | CST | #2391 | 1:1000 for WB |
| Anti-PI3Kα | CST | #4249 | 1:1000 for WB |
| Anti-PI3Kβ | CST | #3011 | 1:1000 for WB |
| Anti-PI3Kγ | CST | #5405 | 1:1000 for WB |
| Anti-β-tubulin | CST | #2128 | 1:1000 for WB |
| Anti-GSK3β | CST | #12456 | 1:1000 for WB |
| Anti-P-GSK3β | CST | #5558 | 1:1000 for WB |
| Anti-p-mTORC1(Ser2481) | CST | #2971 | 1:1000 for WB |
| Anti-mTORC1 | CST | #2972 | 1:1000 for WB |
| Anti-P62 | CST | #23214 | 1:1000 for WB |
| Anti-LC3A/B | CST | #12741 | 1:1000 for WB |
| Anti-S6K | CST | #9202 | 1:1000 for WB |
| Anti-p-S6K | CST | #9208 | 1:1000 for WB |
| Anti-LAMP1 | CST | #15665 | 1:200 for IF |
| Anti-LC3B | Abcam | Ab243506 | 1:200 for IF |
| Anti-Beclin-1 | CST | #3495 | 1:1000 for WB |

Supplementary Table S2. Oligonucleotides used for cloning and qRT-PCR

| **Oligonucleotides** | **Sequences (5′-3′)** | |
| --- | --- | --- |
| **shSIK2** | | |
| Sh1SIK2-F | | GATCCCCGCTAATCATGGCCGGTTAAATTTCAAGAGAATTTAACCGGCCATGATTAGCTTTTTA |
| Sh1SIK2-R | | AGCTTAAAAAGCTAATCATGGCCGGTTAAATTCTCTTGAAATTTAACCGGCCATGATTAGCGGG |
| Sh2SIK2-F | | GATCCCCGCTGGATAACAACATGAATATTTCAAGAGAATATTCATGTTGTTATCCAGCTTTTTA |
| Sh2SIK2-R | | AGCTTAAAAAGCTGGATAACAACATGAATATTCTCTTGAAATATTCATGTTGTTATCCAGCGGG |
| **Real-time RT-PCR** | | |
| β-actin-F | | CCTGGCACCCAGCACAAT |
| β-actin-R | | GGGCCGGACTCGTCATACT |
| GAPDH-F | | CCCACATGGCCTCCAAGGAGTA |
| GAPDH-R | | GTGTACATGGCAACTGTGAGGAGG |
| SIK2-F | | CAACAGCAGACAGGATTTGC |
| SIK2-R | | TGACAGAATATGCCAAAAATGG |
| E-cadherin-F | | GTCTGTCATGGAAGGTGCT |
| E-cadherin-R | | TACGACGTTAGCCTCGTTC |
| ZO-1-F | | AGATTTGGAACTCCCTGAGA |
| ZO-1-R | | GCTTCTGTTACTAGGATCCA |
| Vimentin-F | | CCACGAAGAGAAATCCAGG |
| Vimentin-R | | CAGAGAGGTCAGCAAACTTGG |
| N-cadherin-F | | ATCACAGTGACAGATGTCA |
| N-cadherin-R | | AACGCAGTGTACAGAATCAG |
| Snail-F | | CCTTCTCTAGGCCCTGGCT |
| Snail-R | | AGGTTGGAGCGGGTCAGC |
| β-catenin-F | | GACAGAGTTACTTCACTCTA |
| β-catenin-R | | CATTGGCTCTGTTCTGAAGA |
| PHLPP2-F | | CCAATGAGCAAGGACAGGAT |
| PHLPP2-R | | GGTCCTCTGGTTCCATCTGA |
| PP2Ac-F | | CATGAGGGTCCAATGTGTGA |
| PP2Ac-R | | CCCAAAGGTGTAACCAGCTC |
| **AKT siRNA** | | |
| Sense | | Cell Signaling Technology #6211 |
| Antisense | | Cell Signaling Technology #6211 |
| **β-catenin siRNA** | | |
| Sense | | UUGUACCGGAGCCCUUCACTT |
| Antisense | | GUGAAGGGCUCCGGUACAATT |
| **PHLPP2 siRNA** | | |
| Sense | | CCUAUUGUCUGGCAUCUAUTT |
| Antisense | | AUAGAUGCCAGACAAUAGGTT |
| **PP2A siRNA** | | |
| Sense | | CCUCUCGGUUUGGGAAUAATT |
| Antisense | | UUAUUCCCAAACCGAGAGGTT |
| **Beclin-1 (BECN1) siRNA** | | |
| Sense | | CCCAGGAGGAAGAGACUAATT |
| Antisense | | UUAGUCUCUUCCUCCUGGGTT |
| **Nontargeting control siRNA** | | |
| Sense | | UUCUUCGAACGUGUCACGUTT |
| Antisense | | ACGUGACACGUUCGGAGAATT |
